# Silver Nanoparticles: Synthesis, Characterization, Properties, Applications, and Therapeutic Approaches

**DOI:** 10.3390/ijms17091534

**Published:** 2016-09-13

**Authors:** Xi-Feng Zhang, Zhi-Guo Liu, Wei Shen, Sangiliyandi Gurunathan

**Affiliations:** 1 College of Biological and Pharmaceutical Engineering, Wuhan Polytechnic University, Wuhan 430023, China; zhangxf9465@163.com (X.-F.Z.); zhiguo_l@126.com (Z.-G.L.); 2 Key Laboratory of Animal Reproduction and Germplasm Enhancement in Universities of Shandong, College of Animal Science and Technology, Qingdao Agricultural University, Qingdao 266109, China; shenwei427@126.com; 3 Department of Stem Cell and Regenerative Biotechnology, Konkuk University, Seoul 143-701, Korea

**Keywords:** silver nanoparticles, synthesis, characterization, applications, mechanisms, cancer therapy

## Abstract

Recent advances in nanoscience and nanotechnology radically changed the way we diagnose, treat, and prevent various diseases in all aspects of human life. Silver nanoparticles (AgNPs) are one of the most vital and fascinating nanomaterials among several metallic nanoparticles that are involved in biomedical applications. AgNPs play an important role in nanoscience and nanotechnology, particularly in nanomedicine. Although several noble metals have been used for various purposes, AgNPs have been focused on potential applications in cancer diagnosis and therapy. In this review, we discuss the synthesis of AgNPs using physical, chemical, and biological methods. We also discuss the properties of AgNPs and methods for their characterization. More importantly, we extensively discuss the multifunctional bio-applications of AgNPs; for example, as antibacterial, antifungal, antiviral, anti-inflammatory, anti-angiogenic, and anti-cancer agents, and the mechanism of the anti-cancer activity of AgNPs. In addition, we discuss therapeutic approaches and challenges for cancer therapy using AgNPs. Finally, we conclude by discussing the future perspective of AgNPs.

## 1. Introduction

Silver nanoparticles (AgNPs) are increasingly used in various fields, including medical, food, health care, consumer, and industrial purposes, due to their unique physical and chemical properties. These include optical, electrical, and thermal, high electrical conductivity, and biological properties [1,2,3]. Due to their peculiar properties, they have been used for several applications, including as antibacterial agents, in industrial, household, and healthcare-related products, in consumer products, medical device coatings, optical sensors, and cosmetics, in the pharmaceutical industry, the food industry, in diagnostics, orthopedics, drug delivery, as anticancer agents, and have ultimately enhanced the tumor-killing effects of anticancer drugs [4]. Recently, AgNPs have been frequently used in many textiles, keyboards, wound dressings, and biomedical devices [2,5,6]. Nanosized metallic particles are unique and can considerably change physical, chemical, and biological properties due to their surface-to-volume ratio; therefore, these nanoparticles have been exploited for various purposes [7,8]. In order to fulfill the requirement of AgNPs, various methods have been adopted for synthesis. Generally, conventional physical and chemical methods seem to be very expensive and hazardous [1,9]. Interestingly, biologically-prepared AgNPs show high yield, solubility, and high stability [1]. Among several synthetic methods for AgNPs, biological methods seem to be simple, rapid, non-toxic, dependable, and green approaches that can produce well-defined size and morphology under optimized conditions for translational research. In the end, a green chemistry approach for the synthesis of AgNPs shows much promise.

After synthesis, precise particle characterization is necessary, because the physicochemical properties of a particle could have a significant impact on their biological properties. In order to address the safety issue to use the full potential of any nano material in the purpose of human welfare, in nanomedicines, or in the health care industry, etc., it is necessary to characterize the prepared nanoparticles before application [10,11]. The characteristic feature of nanomaterials, such as size, shape, size distribution, surface area, shape, solubility, aggregation, etc. need to be evaluated before assessing toxicity or biocompatibility [12]. To evaluate the synthesized nanomaterials, many analytical techniques have been used, including ultraviolet visible spectroscopy (UV-vis spectroscopy), X-ray diffractometry (XRD), Fourier transform infrared spectroscopy (FTIR), X-ray photoelectron spectroscopy (XPS), dynamic light scattering (DLS), scanning electron microscopy (SEM), transmission electron microscopy (TEM), atomic force microscopy (AFM), and so on [13,14].

The biological activity of AgNPs depends on factors including surface chemistry, size, size distribution, shape, particle morphology, particle composition, coating/capping, agglomeration, and dissolution rate, particle reactivity in solution, efficiency of ion release, and cell type, and the type of reducing agents used for the synthesis of AgNPs are a crucial factor for the determination of cytotoxicity [15]. The physicochemical properties of nanoparticles enhance the bioavailability of therapeutic agents after both systemic and local administration [16,17] and other hand it can affect cellular uptake, biological distribution, penetration into biological barriers, and resultant therapeutic effects [18,19]. Therefore, the development of AgNPs with controlled structures that are uniform in size, morphology, and functionality are essential for various biomedical applications [20,21,22,23,24].

Cancer is a complex, multifactorial disease which has the characteristic feature of the uncontrolled growth and spread of abnormal cells caused by several factors, including a combination of genetic, external, internal, and environmental factors [25], and it is treated by various treatments including chemotherapy, hormone therapy, surgery, radiation, immune therapy, and targeted therapy [25]. Therefore, the challenge is to identify effective, cost-effective, and sensitive lead molecules that have cell-targeted specificity and increase the sensitivity. Recently, AgNPs have been shown much interest because of their therapeutic applications in cancer as anticancer agents, in diagnostics, and in probing. Taken literature into consideration, in this review we focused on recent developments in synthesis, characterization, properties, and bio-applications mainly on the antibacterial, antifungal, antiviral, anti-inflammatory, anti-cancer and anti-angiogenic properties of AgNPs in a single platform. This review also emphasizes mechanism of anticancer activity, therapeutic approaches and the challenges and limitations of nanoparticles in cancer therapy. Finally, this review ends with conclusion and the future perspective of AgNPs.

## 2. Synthesis of AgNPs

### 2.1. Synthesis of AgNPs Using Physical and Chemical Methods

Generally, the synthesis of nanoparticles has been carried out using three different approaches, including physical, chemical, and biological methods. In physical methods, nanoparticles are prepared by evaporation-condensation using a tube furnace at atmospheric pressure [26,27,28,29]. Conventional physical methods including spark discharging and pyrolysis were used for the synthesis of AgNPs [30,31]. The advantages of physical methods are speed, radiation used as reducing agents, and no hazardous chemicals involved, but the downsides are low yield and high energy consumption, solvent contamination, and lack of uniform distribution [32,33,34,35,36].

Chemical methods use water or organic solvents to prepare the silver nanoparticles [37,38]. This process usually employs three main components, such as metal precursors, reducing agents, and stabilizing/capping agents. Basically, the reduction of silver salts involves two stages (1) nucleation; and (2) subsequent growth. In general, silver nanomaterials can be obtained by two methods, classified as “top-down” and “bottom-up” [39]. The “top-down” method is the mechanical grinding of bulk metals with subsequent stabilization using colloidal protecting agents [40,41]. The “bottom-up” methods include chemical reduction, electrochemical methods, and sono-decomposition. The major advantage of chemical methods is high yield, contrary to physical methods, which have low yield. The above-mentioned methods are extremely expensive. Additionally, the materials used for AgNPs synthesis, such as citrate, borohydride, thio-glycerol, and 2-mercaptoethanol are toxic and hazardous [41]. Apart from these disadvantages, the manufactured particles are not of expected purity, as their surfaces were found to be sedimented with chemicals. It is also very difficult to prepare AgNPs with a well-defined size, requiring a further step for the prevention of particle aggregation [42]. In addition, during the synthesis process, too many toxic and hazardous byproducts are excised out. Chemical methods make use of techniques such as cryochemical synthesis [43], laser ablation [44], lithography [45], electrochemical reduction [46], laser irradiation [47], sono-decomposition [48], thermal decomposition [49], and chemical reduction [50]. The advantage of the chemical synthesis of nanoparticles are the ease of production, low cost, and high yield; however, the use of chemical reducing agents are harmful to living organisms [13]. Recently, Abbasi et al. explained a detailed account of synthesis methods, properties, and bio-application of AgNPs [51].

### 2.2. Green Chemistry Approach for the Synthesis of AgNPs

To overcome the shortcomings of chemical methods, biological methods have emerged as viable options. Recently, biologically-mediated synthesis of nanoparticles have been shown to be simple, cost effective, dependable, and environmentally friendly approaches and much attention has been given to the high yield production of AgNPs of defined size using various biological systems including bacteria, fungi, plant extracts, and small biomolecules like vitamins and amino acids as an alternative method to chemical methods—not only for AgNPs, but also for the synthesis of several other nanoparticles, such as gold and graphene [9,52,53,54,55,56]. Bio-sorption of metals by Gram-negative and Gram-positive bacteria provided an indication for the synthesis of nanoparticles before the flourishing of this biological method; however, the synthesized nanomaterials were as aggregates not nanoparticles [57]. Several studies reported the synthesis of AgNPs using green, cost effective, and biocompatible methods without the use of toxic chemicals in biological methods. In this green chemistry approach, several bacteria, including *Pseudomonas stutzeri* AG259 [58], *Lactobacillus* strains [59], *Bacillus licheniformis* [55]; *Escherichia coli* (*E. coli*) [9], *Brevibacterium casei* [60], fungi including *Fusarium oxysporum* [61], *Ganoderma neo-japonicum* Imazeki [62], plant extracts such as *Allophylus cobbe* [52], *Artemisia princeps* [63], and *Typha angustifolia* [64] were utilized. In addition to these, several biomolecules, such as biopolymers [65], starch [66], fibrinolytic enzyme [39], and amino acids [67] were used. The biological synthesis of nanoparticles depends on three factors, including (a) the solvent; (b) the reducing agent; and (c) the non-toxic material. The major advantage of biological methods is the availability of amino acids, proteins, or secondary metabolites present in the synthesis process, the elimination of the extra step required for the prevention of particle aggregation, and the use of biological molecules for the synthesis of AgNPs is eco-friendly and pollution-free. Biological methods seem to provide controlled particle size and shape, which is an important factor for various biomedical applications [68]. Using bacterial protein or plant extracts as reducing agents, we can control the shape, size, and monodispersity of the nanoparticles [9]. The other advantages of biological methods are the availability of a vast array of biological resources, a decreased time requirement, high density, stability, and the ready solubility of prepared nanoparticles in water [69].

The biological activity of AgNPs depends on the morphology and structure of AgNPs, controlled by size and shape of the particles [70,71]. As far as size and shape are concerned, smaller size and truncated-triangular nanoparticles seem to be more effective and have superior properties. Although many studies successfully synthesized AgNPs with different shape and size ranges, they still have certain limitations. To achieve control over morphology and structure, an excess of strong reducing agent such as sodium borohydride (NaBH_4_) was used for the synthesis of monodisperse and uniform-sized silver colloids [72]. Compared to chemical methods, biological methods allow for more ease in the control of shape, size, and distribution of the produced nanoparticles by optimization of the synthesis methods, including the amount of precursors, temperature, pH, and the amount of reducing and stabilizing factors [9,73].

## 3. Characterization

**Figure 1:**
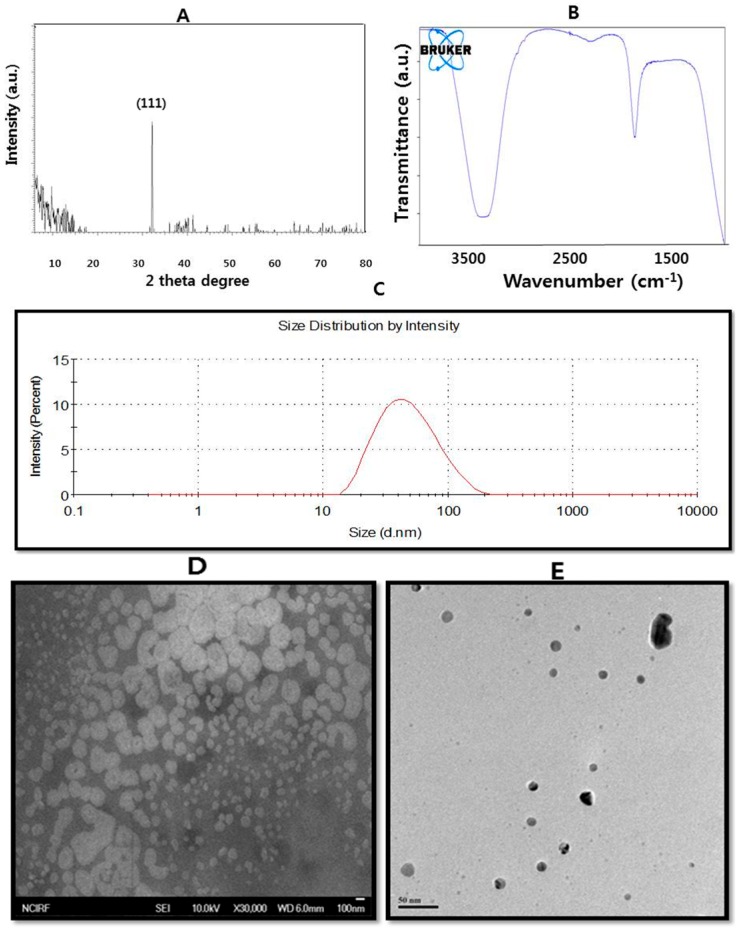
Characterization of silver nanoparticles (AgNPs) prepared from *Bacillus* species using various analytical techniques. (**A**) Characterization of AgNPs by X-diffraction spectra of AgNPs; (**B**) Fourier transform infrared spectra of AgNPs; (**C**) Measurement of size distribution of AgNPs by dynamic light scattering; (**D**) Scanning electron microscopy images of AgNPs; (**E**). Transmission electron microscopy images of AgNPs.

The physicochemical properties of nanoparticles are important for their behavior, bio-distribution, safety, and efficacy. Therefore, characterization of AgNPs is important in order to evaluate the functional aspects of the synthesized particles. Characterization is performed using a variety of analytical techniques, including UV-vis spectroscopy, X-ray diffractometry (XRD), Fourier transform infrared spectroscopy (FTIR), X-ray photoelectron spectroscopy (XPS), dynamic light scattering (DLS), scanning electron microscopy (SEM), transmission electron microscopy (TEM), and atomic force microscopy (AFM). Several qualified books and reviews have presented the principles and usage of various kinds of analytical techniques for the characterization of AgNPs; however, the basics of the important techniques used for the characterization of AgNPs are detailed below for ease of understanding. For example, characterization of AgNPs using various analytical techniques prepared from culture supernatant of *Bacillus* species was given in Figure 1.

### 3.1. UV-Visible Spectroscopy

UV-vis spectroscopy is a very useful and reliable technique for the primary characterization of synthesized nanoparticles which is also used to monitor the synthesis and stability of AgNPs [74]. AgNPs have unique optical properties which make them strongly interact with specific wavelengths of light [75]. In addition, UV-vis spectroscopy is fast, easy, simple, sensitive, selective for different types of NPs, needs only a short period time for measurement, and finally a calibration is not required for particle characterization of colloidal suspensions [76,77,78]. In AgNPs, the conduction band and valence band lie very close to each other in which electrons move freely. These free electrons give rise to a surface plasmon resonance (SPR) absorption band, occurring due to the collective oscillation of electrons of silver nano particles in resonance with the light wave [79,80,81,82,83,84]. The absorption of AgNPs depends on the particle size, dielectric medium, and chemical surroundings [81,82,83,84,85]. Observation of this peak—assigned to a surface plasmon—is well documented for various metal nanoparticles with sizes ranging from 2 to 100 nm [74,86,87]. The stability of AgNPs prepared from biological methods was observed for more than 12 months, and an SPR peak at the same wavelength using UV-vis spectroscopy was observed.

### 3.2. X-ray Diffraction (XRD)

X-ray diffraction (XRD) is a popular analytical technique which has been used for the analysis of both molecular and crystal structures [79,88], qualitative identification of various compounds [89], quantitative resolution of chemical species [90], measuring the degree of crystallinity [91], isomorphous substitutions [92], particle sizes [93], etc. When X-ray light reflects on any crystal, it leads to the formation of many diffraction patterns, and the patterns reflect the physico-chemical characteristics of the crystal structures. In a powder specimen, diffracted beams typically come from the sample and reflect its structural physico-chemical features. Thus, XRD can analyze the structural features of a wide range of materials, such as inorganic catalysts, superconductors, biomolecules, glasses, polymers, and so on [94]. Analysis of these materials largely depends on the formation of diffraction patterns. Each material has a unique diffraction beam which can define and identify it by comparing the diffracted beams with the reference database in the Joint Committee on Powder Diffraction Standards (JCPDS) library. The diffracted patterns also explain whether the sample materials are pure or contain impurities. Therefore, XRD has long been used to define and identify both bulk and nanomaterials, forensic specimens, industrial, and geochemical sample materials [95,96,97,98,99,100,101,102,103,104].

XRD is a primary technique for the identification of the crystalline nature at the atomic scale [10,14,88,105]. X-ray powder diffraction is a nondestructive technique with great potential for the characterization of both organic and inorganic crystalline materials [106]. This method has been used to measure phase identification, conduct quantitative analysis, and to determine structure imperfections in samples from various disciplines, such as geological, polymer, environmental, pharmaceutical, and forensic sciences. Recently, the applications have extended to the characterization of various nano-materials and their properties [106]. The working principle of X-ray diffraction is Bragg’s law [88,105]. Typically, XRD is based on the wide-angle elastic scattering of X-rays [10,14,88,107,108,109]. Although XRD has several merits, it has limited disadvantages, including difficulty in growing the crystals and the ability to get results pertaining only to single conformation/binding state [14,108,110]. Another drawback of XRD is the low intensity of diffracted X-rays compared to electron diffractions [110,111].

### 3.3. Dynamic Light Scattering

Physicochemical characterization of prepared nanomaterials is an important factor for the analysis of biological activities using radiation scattering techniques [10,14,112]. DLS can probe the size distribution of small particles a scale ranging from submicron down to one nanometer in solution or suspension [10,14,113]. Dynamic light scattering is a method that depends on the interaction of light with particles. This method can be used for the measurement of narrow particle size distributions, especially in the range of 2–500 nm [78]. Among the techniques for the characterization of nanoparticles, the most commonly used is DLS [114,115,116]. DLS measures the light scattered from a laser that passes through a colloid, and mostly relies on Rayleigh scattering from the suspended nanoparticles [117]. Next, the modulation of the scattered light intensity as a function of time is analyzed, and the hydrodynamic size of particles can be determined [118,119,120]. To evaluate the toxic potential of any nanomaterial, its characterization in solution is essential [11]. Therefore; DLS is mainly used to determine particle size and size distributions in aqueous or physiological solutions [12]. The size obtained from DLS is usually larger than TEM, which may be due to the influence of Brownian motion. DLS is a nondestructive method used to obtain the average diameter of nanoparticles dispersed in liquids. It has the special advantage of probing a large quantity of particles simultaneously; however, it has a number of sample-specific limitations [101,121].

### 3.4. Fourier Transform Infrared (FTIR) Spectroscopy

FTIR is able to provide accuracy, reproducibility, and also a favorable signal-to-noise ratio. By using FTIR spectroscopy, it becomes possible to detect small absorbance changes on the order of 10^−3^, which helps to perform difference spectroscopy, where one could distinguish the small absorption bands of functionally active residues from the large background absorption of the entire protein [122,123,124,125,126,127,128]. FTIR spectroscopy is frequently used to find out whether biomolecules are involved in the synthesis of nanoparticles, which is more pronounced in academic and industrial research [10,68,129,130]. Furthermore, FTIR has also been extended to the study of nano-scaled materials, such as confirmation of functional molecules covalently grafted onto silver, carbon nanotubes, graphene and gold nanoparticles, or interactions occurring between enzyme and substrate during the catalytic process [68,131,132]. Furthermore, it is a non-invasive technique. Finally, the advantages of FTIR spectrometers over dispersive ones are rapid data collection, strong signal, large signal-to-noise ratio, and less sample heat-up [133]. Recently, further advancement has been made in an FTIR method called attenuated total reflection (ATR)-FTIR spectroscopy [134,135,136]. Using ATR-FTIR, we can determine the chemical properties on the polymer surface, and sample preparation is easy compared to conventional FTIR [10,137,138,139,140,141]. Therefore, FTIR is a suitable, valuable, non-invasive, cost effective, and simple technique to identify the role of biological molecules in the reduction of silver nitrate to silver.

### 3.5. X-ray Photoelectron Spectroscopy (XPS)

XPS is a quantitative spectroscopic surface chemical analysis technique used to estimate empirical formulae [109,140,141,142]. XPS is also known as electron spectroscopy for chemical analysis (ESCA), [141]. XPS plays a unique role in giving access to qualitative, quantitative/semi-quantitative, and speciation information concerning the sensor surface [143]. XPS is performed under high vacuum conditions. X-ray irradiation of the nanomaterial leads to the emission of electrons, and the measurement of the kinetic energy and the number of electrons escaping from the surface of the nanomaterials gives XPS spectra [109,140,141,142]. The binding energy can be calculated from kinetic energy. Specific groups of starburst macromolecules such as P=S, aromatic rings, C–O, and C=O can be identified and characterized by XPS [144].

### 3.6. Scanning Electron Microscopy

Recently, the field of nanoscience and nanotechnology has provided a driving force in the development of various high-resolution microscopy techniques in order to learn more about nanomaterials using a beam of highly energetic electrons to probe objects on a very fine scale [145,146,147]. Among various electron microscopy techniques, SEM is a surface imaging method, fully capable of resolving different particle sizes, size distributions, nanomaterial shapes, and the surface morphology of the synthesized particles at the micro and nanoscales [10,117,137,148,149]. Using SEM, we can probe the morphology of particles and derive a histogram from the images by either by measuring and counting the particles manually, or by using specific software [117]. The combination of SEM with energy-dispersive X-ray spectroscopy (EDX) can be used to examine silver powder morphology and also conduct chemical composition analysis. The limitation of SEM is that it is not able to resolve the internal structure, but it can provide valuable information regarding the purity and the degree of particle aggregation. The modern high-resolution SEM is able to identify the morphology of nanoparticles below the level of 10 nm.

### 3.7. Transmission Electron Microscopy

TEM is a valuable, frequently used, and important technique for the characterization of nanomaterials, used to obtain quantitative measures of particle and/or grain size, size distribution, and morphology [10,109,150]. The magnification of TEM is mainly determined by the ratio of the distance between the objective lens and the specimen and the distance between objective lens and its image plane [150]. TEM has two advantages over SEM: it can provide better spatial resolution and the capability for additional analytical measurements [10,148,150]. The disadvantages include a required high vacuum, thin sample section [10,109,148], and the vital aspect of TEM is that sample preparation is time consuming. Therefore, sample preparation is extremely important in order to obtain the highest-quality images possible.

### 3.8. Atomic Force Microscopy

Generally, AFM is used to investigate the dispersion and aggregation of nanomaterials, in addition to their size, shape, sorption, and structure; three different scanning modes are available, including contact mode, non-contact mode, and intermittent sample contact mode [10,14,151,152,153,154,155]. AFM can also be used to characterize the interaction of nanomaterials with supported lipid bilayers in real time, which is not achievable with current electron microscopy (EM) techniques [113]. In addition, AFM does not require oxide-free, electrically conductive surfaces for measurement, does not cause appreciable damage to many types of native surfaces, and it can measure up to the sub-nanometer scale in aqueous fluids [156,157]. However, a major drawback is the overestimation of the lateral dimensions of the samples due to the size of the cantilever [158,159]. Therefore, we have to provide much attention to avoid erroneous measurements [160]. Furthermore, the choice of operating mode—no contact or contact—is a crucial factor in sample analysis [160].

### 3.9. Localized Surface Plasmon Resonance (LSPR)

LSPR is a coherent, collective spatial oscillation of the conduction electrons in a metallic nanoparticle, which can be directly excited by near-visible light. The localized surface plasmon resonance (LSPR) condition is defined by several factors, including the electronic properties of the nanoparticle, the size and shape of the particle, temperature, the dielectric environment, and so on. Small changes in the local dielectric environment cause the dysfunction of LSPR. The frequency of the LSPR spectral peak is very sensitive to the nanostructure environment through the local refractive index. Thereby, shifts of the LSPR frequency are widely used as a method for the detection of molecular interaction close to the surface of the nanoparticle [161,162,163,164,165,166]. In addition, the near-field enhancement has led to a very large variety of advances in many fundamental and applied areas of science, particularly for the determination of nanoparticle shapes, dimensions, and compositions. This spectroscopy method is being used to investigate fundamental properties and processes of nanoparticles in (bio)-molecular detection devices, or (bio)-imaging tools with improved single-molecule sensitivity. LSPR spectroscopy can provide thermodynamic and real-time kinetic data for binding processes. LSPR-based tools will be helpful to analyze faster and with higher sensitivity. The application of LSPR spectroscopy is mainly used for biological and chemical sensing by transducing changes in the local refractive index via a wavelength-shift measurement, due to its sensitivity, wavelength tunability, smaller sensing volumes, and lower instrumentation cost. Single-nanoparticle LSPR spectroscopy is an important tool for understanding the relationship between local structure and spectra. In addition, single nanoparticles can provide even higher refractive-index sensitivity than nanoparticle arrays.

## 4. Properties of AgNPs

**Figure 2:**
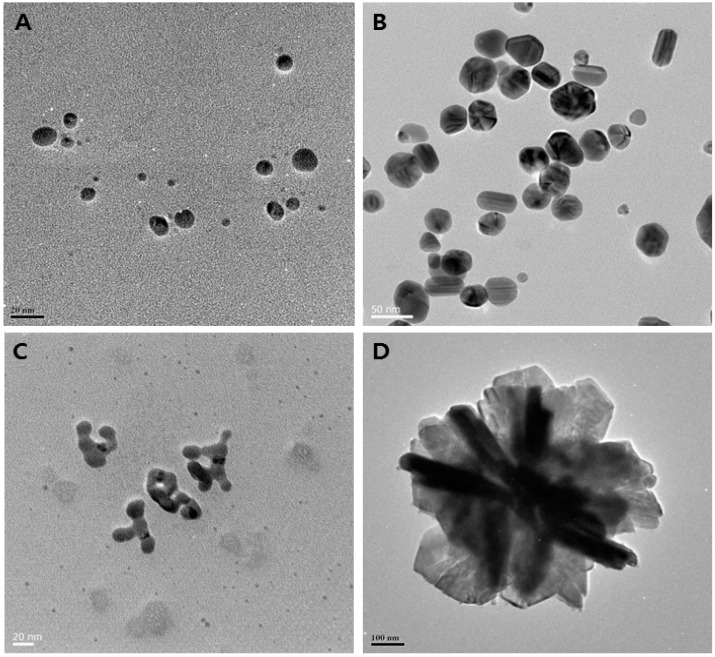
Biological synthesis of various shapes of AgNPs using culture supernatant of various *Bacillus* species. (**A**) Spherical; (**B**) mixed populations (octagonal, rod, hexagonal, and icosahedral); (**C**) highly branched; (**D**) flower-like in shape.

Physical and chemical properties of AgNPs—including surface chemistry, size, size distribution, shape, particle morphology, particle composition, coating/capping, agglomeration, dissolution rate, particle reactivity in solution, efficiency of ion release, cell type, and finally type of reducing agents used for synthesis—are crucial factors for determination of cytotoxicity [15,50,167,168,169,170,171,172,173,174,175,176]. For example, using biological reducing agents such as culture supernatants of various *Bacillus* species, AgNPs can be synthesized in various shapes, such as spherical, rod, octagonal, hexagonal, triangle, flower-like, and so on (Figure 2). Previous studies supported the assertion that smaller size particles could cause more toxicity than larger, because they have larger surface area [176]. Shape is equally important to the determination of toxicity [177]. For example, in the biomedical field, various types of nanostructures have been used, including nanocubes, nanoplates, nanorods, spherical nanoparticles, flower-like, and so on [175,178]. AgNP toxicity mainly depends on the availability of chemical and or biological coatings on the nanoparticle surface [179]. AgNP surface charges could determine the toxicity effect in cells. For instance, the positive surface charge of these NPs renders them more suitable, allowing them to stay for a long time in blood stream compared to negatively-charged NPs [180], which is a major route for the administration of anticancer agents [181,182].

## 5. Biological Applications of AgNPs

**Figure 3:**
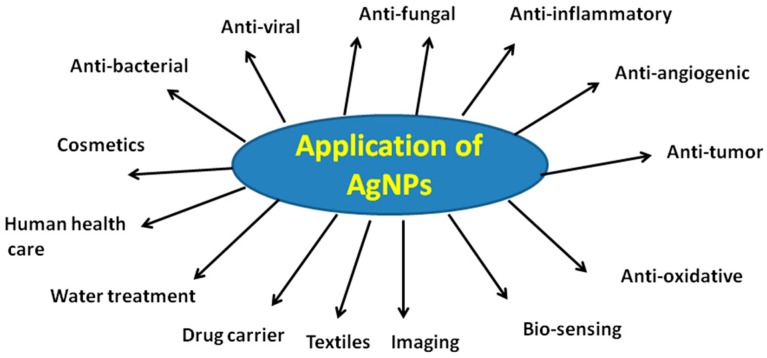
Various applications of AgNPs.

Due to their unique properties, AgNPs have been used extensively in house-hold utensils, the health care industry, and in food storage, environmental, and biomedical applications. Several reviews and book chapters have been dedicated in various areas of the application of AgNPs. Herein, we are interested in emphasizing the applications of AgNPs in various biological and biomedical applications, such as antibacterial, antifungal, antiviral, anti-inflammatory, anti-cancer, and anti-angiogenic. Herein, we specifically addressed previously-published seminal papers and end with recent updates. A schematic diagram representing various applications of AgNPs is provided in Figure 3.

### 5.1. Antibacterial Activity of AgNPs

AgNPs seem to be alternative antibacterial agents to antibiotics and have the ability to overcome the bacterial resistance against antibiotics. Therefore, it is necessary to develop AgNPs as antibacterial agents. Among the several promising nanomaterials, AgNPs seem to be potential antibacterial agents due to their large surface-to-volume ratios and crystallographic surface structure. The seminal paper reported by Sondi and Salopek-Sondi [6] demonstrated the antimicrobial activity of AgNPs against *Escherichia coli*, in which *E. coli* cells treated with AgNPs showed the accumulation of AgNPs in the cell wall and the formation of “pits” in the bacterial cell walls, eventually leading to cell death. In the same *E. coli* strain, smaller particles with a larger surface-to-volume ratio showed a more efficient antibacterial activity than larger particles [183]. Furthermore, the antibacterial activity of AgNPs is not only size—but also shape-dependent [70]. AgNPs were synthesized by four different types of saccharides with an average size of 25 nm, showing high antimicrobial and bactericidal activity against Gram-positive and Gram-negative bacteria, including highly multi-resistant strains such as methicillin-resistant *Staphylococcus aureus*. As mentioned previously, not only the size is important for determining the efficiency, but also shape, because AgNPs undergo a shape-dependent interaction with the Gram-negative organism *E. coli* [71]. Furthermore, a detailed study was carried out to investigate the efficiency of the antimicrobial effects of AgNPs against yeast, *E. coli*, and *Staphylococcus aureus*. The results suggest that at low concentrations of AgNPs, the complete inhibition of growth was observed in yeast and *E. coli*, whereas a mild effect was observed in *S. aureus* [184]. Biologically synthesized AgNPs from the culture supernatants of *Klebsiella pneumoniae* were evaluated; the efficiencies of various antibiotics, such as penicillin G, amoxicillin, erythromycin, clindamycin, and vancomycin against *Staphylococcus aureus* and *E. coli* were increased in the presence of Ag-NPs [185]. When compared to AgNPs, hydrogel–silver nanocomposites showed excellent antibacterial activity against *E. coli*. One-pot synthesis of chitosan–Ag–nanoparticle composite was found to have higher antimicrobial activity than its components at their respective concentrations, because one-pot synthesis favors the formation of small AgNPs attached to the polymer, which can be dispersed in media of pH ≤ 6.3 [186]. Biologically produced AgNPs using culture supernatants of *Staphylococcus aureus* showed significant antimicrobial activity against methicillin-resistant *S. aureus*, followed by methicillin-resistant *Staphylococcus epidermidis* and *Streptococcus pyogenes*, whereas only moderate antimicrobial activity was observed against *Salmonella typhi* and *Klebsiella pneumoniae* [187]. The mechanisms of AgNP-induced cell death was observed in *E. coli* through the leakage of reducing sugars and proteins. Furthermore, AgNPs are able to destroy the permeability of the bacterial membranes via the generation of many pits and gaps, indicating that AgNPs could damage the structure of the bacterial cell membrane [2]. Silver nanocrystalline chlorhexidine (AgCHX) complex showed strong antibacterial activity against the tested Gram-positive/negative and methicillin-resistant *Staphylococcus aureus* (MRSA) strains. Interestingly, the minimal inhibitory concentrations (MICs) of nanocrystalline Ag(III)CHX were much lower than those of the ligand (CHX), AgNO_3_, and the gold standard, silver sulfadiazine [188].

**Figure 4:**
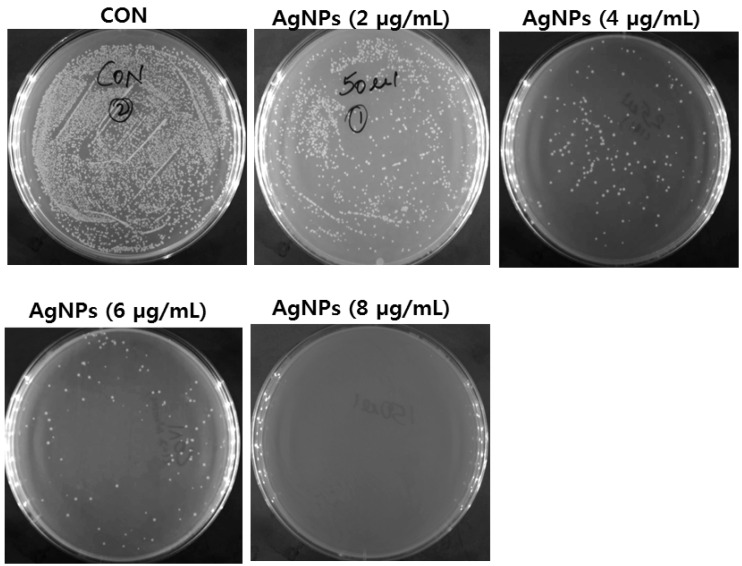
Dose-dependent antibacterial activity of biologically synthesized AgNPs in *E. coli.* CON: control.

Biofilms are not only leads to antimicrobial resistance, but are involved in the development of ocular-related infectious diseases, such as microbial keratitis [189]. Kalishwaralal and co-workers demonstrated the potential anti-biofilm activity against *Pseudomonas aeruginosa* and *Staphylococcus epidermidis*. Similarly, guava leaf extract reduced AgNPs (Gr-Ag-NPs) showed significant antibacterial activity and stability against *E. coli* compared to chemically synthesized AgNPs; the reason for this higher activity could be the adsorption of biomolecules on the surface of the Gr-Ag-NPs [190]. AgNPs synthesized by *Cryphonectria* sp. showed antibacterial activity against various human pathogenic bacteria, including *S. aureus*, *E. coli*, *Salmonella typhi*, and *Candida albicans*. Interestingly, these particular AgNPs exhibited higher antibacterial activity against both *S. aureus* and *E. coli* than against *S. typhi* and *C. albicans*. Figure 4 shows the effectiveness of dose-dependent antibacterial activity of biologically synthesized AgNPs in *E. coli.*

**Figure 5:**
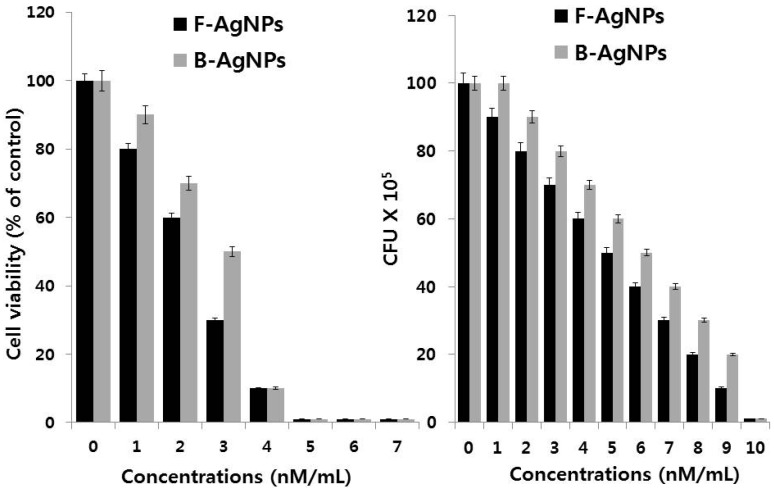
Differential antibacterial activity of AgNPs synthesized with *Calocybe indica* extracts (F-AgNPs) and the culture supernatant of *Bacillus tequilensis* (B-AgNPs) as reducing agents.

Besinis et al. [191] compared the toxic efficiency of different nanomaterials, such as AgNPs, silver, and titanium dioxide against routine disinfectant chlorhexidine in *Streptococcus mutans*. Among various nanomaterials, AgNPs had the strongest antibacterial activity of the NPs tested. Agnihotri et al. [192] demonstrated that the mechanisms of AgNPs on bactericidal action using AgNPs immobilized on an amine-functionalized silica surface. They found that contact killing is the predominant bactericidal mechanism, and surface-immobilized nanoparticles show greater efficacy than colloidal AgNPs, as well as a higher concentration of silver ions in solution. The nanocomposite containing silver/polyrhodanine nanocomposite-decorated silica nanoparticles shows potential and enhanced antibacterial activity against *E. coli* and *S. aureus*, which is due to the particular combination of AgNPs and the polyrhodanine [155]. Interestingly, Khurana et al. [193] investigated the antimicrobial properties of silver based on its physical and surface properties against *S. aureus*, *B. megaterium*, *P. vulgaris*, and *S. sonnei*. The enhancement of antibacterial action was observed with particles having a hydrodynamic size of 59 nm compared to 83 nm. Gurunathan et al. [68] reported that the antibacterial and anti-biofilm activity of antibiotics, AgNPs, or combinations of AgNPs against important pathogenic bacteria *Pseudomonas aeruginosa*, *Shigella flexneri*, *Staphylococcus aureus*, and *Streptococcus pneumoniae*. The results suggest that, the combination of both antibiotics and AgNPs showed significant antimicrobial and anti-biofilm effects at the lowest concentration of antibiotics and AgNPs compared to AgNPs or antibiotics alone. Nanocomposite spheres composed of AgNPs decorated on the polymer colloids exhibited excellent antibacterial activity [194]. Recently, nanocomposites containing graphene and AgNPs showed much interest in antibacterial activity. Graphene oxide (GO)-Ag nanocomposite showed enhanced antibacterial activity against *E. coli* and *S. aureus* using the conventional plate count method and disk diffusion method [195]. The GO-Ag nanocomposite exhibited an excellent antibacterial activity against methicillin-resistant *S. aureus*, *Acinetobacter baumannii*, *Enterococcus faecalis*, and *Escherichia coli*. In addition, GO-Ag nanocomposite is a promising antibacterial agent against common nosocomial bacteria, particularly antibiotic-resistant MRSA [196]. AgNPs derived from fungal extracts as reducing agents (F-AgNPs) showed enhanced antibacterial activity both in *Pseudomonas aeruginosa* and *Staphylococcus aureus* when compared to AgNPs derived from the culture supernatant of bacteria (B-AgNPs) (Figure 5). The minimum inhibitory concentration of F-AgNPs is lesser than B-AgNPs. Nano-silver interacts with peptides and bacteria and serves as nanomedicine in various bacteria, fungi, and virus-mediated diseases [197].

### 5.2. Antifungal Activity of AgNPs

Fungal infections are more frequent in patients who are immunosuppressed, and overcoming fungi-mediated diseases is a tedious process, because currently there is a limited number of available antifungal drugs [198]. Therefore, there is an inevitable and urgent need to develop antifungal agents, which should be biocompatible, non-toxic, and environmentally friendly. At this juncture, AgNPs play an important role as anti-fungal agents against various diseases caused by fungi. Nano-Ag showed potent anti-fungal activity against clinical isolates and ATCC strains of *Trichophyton mentagrophytes* and *Candida species* with concentrations of 1–7 μg/mL. Esteban-Tejeda et al. [199] developed an inert matrix containing AgNPs with an average size of 20 nm into a soda-lime glass which shows enhanced biocidal activity. Monodisperse Nano-Ag sepiolite fibers showed significant antifungal activity against *Issatchenkia* *orientalis*. AgNPs exhibited good antifungal activity against *Aspergillus niger* and a MIC of 25 μg/mL against *Candida albicans* [200]. Biologically-synthesized AgNPs showed enhanced antifungal activity with fluconazole against *Phoma glomerata*, *Phoma herbarum*, *Fusarium semitectum*, *Trichoderma* sp., and *Candida albicans* [201]. AgNPs stabilized by sodium dodecyl sulfate showed enhanced antifungal activity against *Candida albicans* compared to conventional antifungal agents [20]. The size-dependent antifungal activities of different AgNPs were performed against mature *Candida albicans* and *Candida glabrata* biofilms. Biologically synthesized AgNPs exhibited antifungal activity against several phytopathogenic fungi, including *Alternaria alternata*, *Sclerotinia sclerotiorum*, *Macrophomina phaseolina*, *Rhizoctonia solani*, *Botrytis cinerea*, and *Curvularia lunata* at the concentration of 15 mg [202,203]. Similarly, The AgNPs synthesized by *Bacillus* species exhibited strong antifungal activity against the plant pathogenic fungus *Fusarium oxysporum* at the concentration of 8 μg/mL [204]. Carbon nanoscrolls (CNSs) composed of graphene oxides and AgNPs exhibited enhanced and prolonged antifungal activity against *Candida albicans* and *Candida tropical* compared to GO–AgNP nanocomposites containing graphene oxide and AgNPs [205]. The antifungal efficacy of AgNPs was evaluated in combination with nystatin (NYT) or chlorhexidine (CHX) against *Candida albicans* and *Candida glabrata* biofilms. The results from this investigation suggest that AgNPs combined with either nystatin (NYT) or chlorhexidine digluconate (CHG) showed better synergistic anti-biofilm activity; however, this activity depends on the species and drug concentrations [206].

The biologically synthesized AgNPs exhibited strong antifungal activity against *Bipolaris sorokiniana* by the inhibition of conidial germination [207]. Interestingly, AgNPs not only inhibit human and plant pathogenic fungi, but also indoor fungal species such as *Penicillium brevicompactum*, *Aspergillus fumigatus*, *Cladosporium cladosporoides*, *Chaetomium globosum*, *Stachybotrys chartarum*, and *Mortierella alpine* cultured on agar media [208].

### 5.3. Antiviral Activity of AgNPs

Viral mediated diseases are common and becoming more prominent in the world; therefore, developing anti-viral agents is essential. The mechanisms of the antiviral activity of AgNPs are an important aspect in antiviral therapy. AgNPs have unique interactions with bacteria and viruses based on certain size ranges and shapes [70,209,210]. The antiviral activity nano-Ag incorporated into polysulfone ultrafiltration membranes (nAg-PSf) was evaluated against MS2 bacteriophage, which shows that significant antiviral activity was a result of increased membrane hydrophilicity [21]. Lara et al. [211] showed the first mechanistic study demonstrating anti-HIV activity at an early stage of viral replication. Poly vinyl pyrrolidone (PVP)-coated AgNPs prevented the transmission of cell-associated HIV-1 and cell-free HIV-1 isolates [211]. AgNPs have demonstrated efficient inhibitory activities against human immunodeficiency virus (HIV) and hepatitis B virus (HBV) [212]. A study was attempted to investigate the antiviral action of the AgNPs; the data showed that both macrophage (M)-tropic and T-lymphocyte (T)-tropic strains of HIV-1 were highly sensitive to the AgNP-coated polyurethane condom (PUC) [213]. Although several studies have shown that AgNPs could inhibit the viability of viruses, the exact mechanism of antiviral activity is still obscure. However, the studies from Trefry and Wooley found that AgNPs caused a four- to five-log reduction in viral titer at concentrations that were not toxic to cells [214]. Interestingly, in the presence of AgNPs, virus was capable of adsorbing to cells, and this viral entry is responsible for the antiviral effects of AgNPs. Hemagglutination assay indicated that AgNPs could significantly inhibit the growth of the influenza virus in Madin-Darby canine kidney cells. The study from intranasal AgNP administration in mice significantly enhanced survival, lower lung viral titer levels, minor pathologic lesions in lung tissue, and remarkable survival advantage after infection with the H3N2 influenza virus, suggesting that AgNPs had a significant role in mice survival [215]. Biologically-synthesized AgNPs inhibited the viability in herpes simplex virus (HSV) types 1 and 2 and human parainfluenza virus type 3, based on size and zeta potential of AgNPs [216]. The treatment of Vero cells with non-cytotoxic concentrations of AgNPs significantly inhibited the replication of Peste des petits ruminants virus (PPRV). The mechanisms of viral replication are due to the interaction of AgNPs with the virion surface and the virion core [217]. Tannic acid mediated the synthesis of various sizes of AgNPs capable of reducing HSV-2 infectivity both in vitro and in vivo through direct interaction, blocked virus attachment, penetration, and further spread [218]. The antiviral property of Ag^+^ alone and a combination of 50 ppb Ag^+^ and 20 ppm CO_3_^2−^ (carbonate ions) was performed on *bacteriophage* MS2 phage. The results from this study showed that 50 ppb Ag^+^ alone was unable to affect the phage, and the combination of 50 ppb Ag^+^ and 20 ppm CO_3_ was found to have an effective antiviral property within a contact time of 15 min [219]. Treatment with AgNPs for 24 h in Bean Yellow Mosaic Virus (BYMV) decreased virus concentration, percentage of infection, and disease severity [220].

### 5.4. Anti-Inflammatory Activity of AgNPs

Inflammation is an early immunological response against foreign particles by tissue, which is supported by the enhanced production of pro-inflammatory cytokines, the activation of the immune system, and the release of prostaglandins and chemotactic substances such as complement factors, interleukin-1 (IL-1), TNF-α, and TGF-β [221,222,223,224]. In order to overcome inflammatory action, we need to find effective anti-inflammatory agents. Among several anti-inflammatory agents, AgNPs have recently played an important role in anti-inflammatory field. AgNPs have been known to be antimicrobial, but the anti-inflammatory responses of AgNPs are still limited. Bhol and Schechter [225] reported the anti-inflammatory activity in rat. Rats treated intra-colonically with 4 mg/kg or orally with 40 mg/kg of nanocrystalline silver (NPI 32101) showed significantly reduced colonic inflammation. Mice treated with AgNPs showed rapid healing and improved cosmetic appearance, occurring in a dose-dependent manner. Furthermore, AgNPs showed significant antimicrobial properties, reduction in wound inflammation, and modulation of fibrogenic cytokines [226]. Continuing the previous study, Wong et al. [222] investigated to gain further evidence for the anti-inflammatory properties of AgNPs, in which they used both in vivo and in vitro models and found that AgNPs are able to down-regulate the quantities of inflammatory markers, suggesting that AgNPs could suppress inflammatory events in the early phases of wound healing [222]. A porcine contact dermatitis model showed that treatment with nanosilver significantly increases apoptosis in the inflammatory cells and decreased the levels of pro-inflammatory cytokines [227]. Biologically-synthesized AgNPs can inhibit the production of cytokines induced by UV-B irradiation in HaCaT cells, and also reduces the edema and cytokine levels in the paw tissues [228].

### 5.5. Anti-Angiogenic Activity of AgNPs

Pathological angiogenesis is a symbol of cancer and various ischemic and inflammatory diseases [229]. There are several research groups interested in discovering novel pro- and anti-angiogenic molecules to overcome angiogenic-related diseases. Although there are several synthetic molecules having anti-angiogenic properties, the discovery of a series of natural pro- and anti-angiogenic factors suggests that this may provide a more physiological approach to treat both classes of angiogenesis-dependent diseases in the near future [230]. Recently, several studies provided supporting evidence using both in vitro and in vivo models showing that AgNPs have both anti-angiogenic and anti-cancer properties. Herein, we wish to summarize the important contribution in cancer and other angiogenic related diseases.

**Figure 6:**
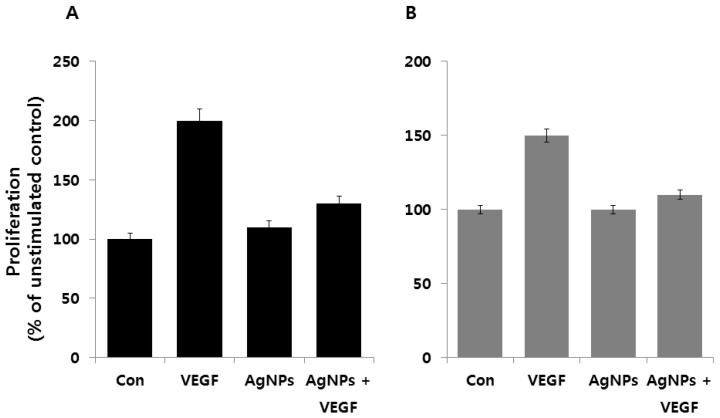
Effect of AgNPs on vascular endothelial growth factor (VEGF)-induced proliferation of (**A**) bovine retinal endothelial cells (BRECs); and (**B**) human breast cancer cells MDA-MB 231. Cells were treated with VEGF with or without AgNPs for 24 h. Cell proliferation was determined by trypan blue exclusion assay.

Kalishwaralal et al. [231] demonstrated the anti-angiogenic property of biologically synthesized AgNPs using bovine retinal endothelial cells (BRECs) as a model system, in which they found the inhibition of proliferation and migration in BRECs after 24 h of treatment with AgNPs at 500 nM concentration. The mechanisms of inhibition of vascular endothelial growth factor (VEGF) induced angiogenic process by the activation of caspase-3 and DNA fragmentation, and AgNPs inhibited the VEGF-induced PI3K/Akt pathway in BRECs [232]. Followed by this study, Gurunathan et al. [23] provided evidence for the anti-angiogenic property of AgNPs by using pigment epithelium derived factor (PEDF) as a bench mark, which is known as a potent anti-angiogenic agent. Using BRECs as an in vitro model system, they found that AgNPs inhibited VEGF-induced angiogenic assays. Furthermore, they demonstrated that AgNPs could block the formation of new blood microvessels by the inactivation of PI3K/Akt. The same group also demonstrated the anticancer property of AgNPs using various cytotoxicity assays in Dalton’s lymphoma ascites (DLA) cells, and a tumor mouse model showed significantly increased survival time in the presence of AgNPs [24]. AgNPs reduced with diaminopyridinyl (DAP)-derivatized heparin (HP) polysaccharides (DAPHP) inhibited basic fibroblast growth factor (FGF-2)-induced angiogenesis compared to glucose conjugation [232]. Kim et al. [233] developed an anti-angiogenic Flt1 peptide conjugated to tetra-*N*-butyl ammonium modified hyaluronate (HA-TBA), and it was used to encapsulate genistein. Using human umbilical vein endothelial cells (HUVECs) as in vitro model system, they found that genistein/Flt1 peptide–HA micelle inhibited the proliferation of HUVECs, and the same reagents could drastically reduce corneal neovascularization in silver nitrate-cauterized corneas of Sprague Dawley (SD) rats. Ag_2_S quantum dots (QDs) used as nanoprobes to monitor lymphatic drainage and vascular networks. Ag_2_S-based nanoprobes showed long circulation time and high stability. In addition, they were able to track angiogenesis mediated by a tiny tumor (2–3 mm in diameter) in vivo [5]. Recently, *Achillea biebersteinii* flowers extract-mediated synthesis of AgNPs containing a concentration of 200 μg/mL showed a 50% reduction in newly-formed vessels [234]. Figure 6 shows the inhibitory effect of AgNPs on VEGF induced angiogenic activity in bovine retinal endothelial cells (BRECs) and human breast cancer cells MDA-MB 231.

### 5.6. Anticancer Activity of AgNPs

In our lifetime, 1 in 3 people has the possibility to develop cancer [235]. Although many chemotherapeutic agents are currently being used on different types of cancers, the side effects are enormous, and particularly, administrations of chemotherapeutic agents by intravenous infusion are a tedious process [235]. Therefore, it is indispensable to develop technologies to avoid systemic side effects. At this juncture, many researchers are interested in developing nanomaterials as an alternative tool to create formulations that can target tumor cells specifically. Several research laboratories have used various cell lines to address the possibility of finding a new molecule to battle cancer. Here we summarized the work from various laboratories reporting anticancer activity using both in vitro and in vivo model systems. Gopinath et al. [236] investigated the molecular mechanism of AgNPs and found that programmed cell death was concentration-dependent under conditions. Further, they observed a synergistic effect on apoptosis using uracil phosphoribosyltransferase (UPRT)-expressing cells and non-UPRT-expressing cells in the presence of fluorouracil (5-FU). In these experimental conditions, they observed that AgNPs not only induce apoptosis but also sensitize cancer cells. The anticancer property of starch-coated AgNPs was studied in normal human lung fibroblast cells (IMR-90) and human glioblastoma cells (U251). AgNPs induced alterations in cell morphology, decreased cell viability and metabolic activity, and increased oxidative stress leading to mitochondrial damage and increased production of reactive oxygen species (ROS), ending with DNA damage. Among these two cell types, U251 cells showed more sensitivity than IMR-90 [237]. The same group also demonstrated that the cellular uptake of AgNPs occurred mainly through endocytosis. AgNP-treated cells exhibited various abnormalities, including upregulation of metallothionein, downregulation of major actin binding protein, filamin, and mitotic arrest [237]. The morphology analysis of cancer cells suggests that biologically synthesized AgNPs could induce cell death very significantly. Jun et al. [238] elegantly prepared multifunctional silver-embedded magnetic nanoparticles, in which the first type consist of silver-embedded magnetic NPs with a magnetic core of average size 18 nm and another type consist of a thick silica shell with silver having an average size of 16 nm; the resulting silica-encapsulated magnetic NPs (M-SERS dots) produce strong surface-enhanced Raman scattering (SERS) signals and have magnetic properties, and these two significant properties were used for targeting breast-cancer cells (SKBR3) and floating leukemia cells (SP2/O).

**Figure 7:**
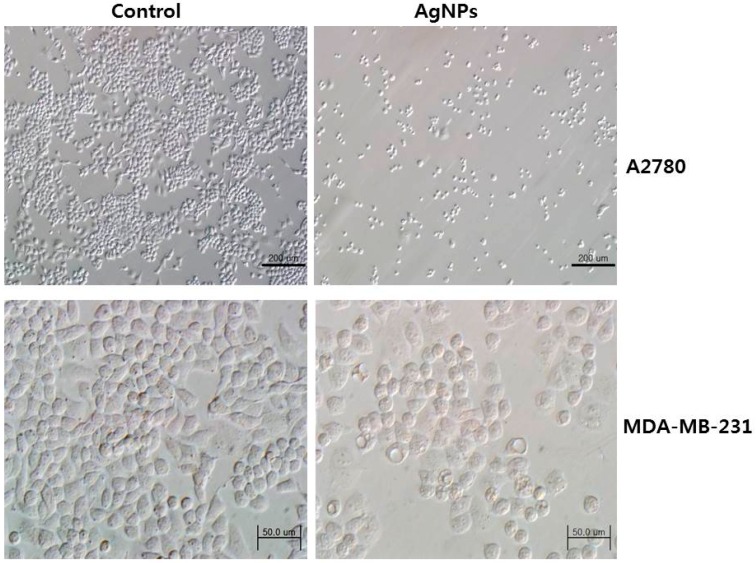
Anticancer activity of biologically synthesized AgNPs using *Bacillus* species in human ovarian cancer and human breast cancer cells.

The antineoplastic activities of protein-conjugated silver sulfide nano-crystals are size dependent in human hepatocellular carcinoma Bel-7402 and C6 glioma cells [239]. Instead of giving direct treatment of AgNPs into the cells, some researchers developed chitosan as a carrier molecule for the delivery of silver to the cancer cells. For example, Sanpui et al. [240] demonstrated that chitosan-based nanocarrier (NC) delivery of AgNPs induces apoptosis at very low concentrations. They then examined cytotoxic efficiency using a battery of biochemical assays. They found an increased level of intracellular ROSin HT 29 cells. Lower concentrations of nanocarrier with AgNPs showed better inhibitory results than AgNPs alone. Boca et al. [241] reported that chitosan-coated silver nanotriangles (Chit-AgNTs) show an increased cell mortality rate. In addition, human embryonic cells (HEK) were able to take up Chit-AgNTs efficiently, and the cytotoxic effect of various sizes of AgNPs was significant in acute myeloid leukemia (AML) cells [242]. Recently, the anticancer property of bacterial (B-AgNPs) and fungal extract-produced AgNPs (F-AgNPs) was demonstrated in human breast cancer MDA-MB-231 cells. Both biologically produced AgNPs exhibited significant cytotoxicity [62,243]. Among these two AgNPs, fungal extract-derived AgNPs had a stronger effect than B-AgNPs, which is due to the type of reducing agents used for the synthesis of AgNPs. Similarly, AgNPs derived from *Escherichia fergusoni* showed dose-dependent cytotoxicity against MCF-7 cells [62]. Plant extract-mediated synthesis of AgNPs showed more pronounced toxic effect in human lung carcinoma cells (A549) than non-cancer cells like human lung cells, indicating that AgNPs could target cell-specific toxicity, which could be the lower level of pH in the cancer cells [63]. Targeted delivery is an essential process for the treatment of cancer. To address this issue, Locatelli et al. [244] developed multifunctional nanocomposites containing polymeric nanoparticles (PNPs) containing alisertib (Ali) and AgNPs. PNPs conjugated with a chlorotoxin (Ali@PNPs–Cltx) showed a nonlinear dose–effect relationship, whereas the toxicity of Ag/Ali@PNPs–Cltx remained stable. Biologically synthesized AgNPs showed significant toxicity in MCF7 and T47D cancer cells by higher endocytic activity than MCF10-A normal breast cell line [245]. Banti and Hadjikakou explained the detailed account of anti-proliferative and anti-tumor activity of silver(I) compounds [246]. Biologically synthesized AgNPs capable of altering cell morphology of cancer cells (Figure 7), which is an early indicator for apoptosis. Apoptosis can be determined by structural alterations in cells by transmitted light microscopy. 

### 5.7. Diagnostic, Biosensor, and Gene Therapy Applications of AgNPs

The advancement in medical technologies is increasing. There is much interest in using nanoparticles to improve or replace today’s therapies. Nanoparticles have advantages over today’s therapies, because they can be engineered to have certain properties or to behave in a certain way. Recent developments in nanotechnology are the use of nanoparticles in the development of new and effective medical diagnostics and treatments. The ability of AgNPs in cellular imaging in vivo could be very useful for studying inflammation, tumors, immune response, and the effects of stem cell therapy, in which contrast agents were conjugated or encapsulated to nanoparticles through surface modification and bioconjugation of the nanoparticles. Silver plays an important role in imaging systems due its stronger and sharper plasmon resonance. AgNPs, due to their smaller size, are mainly used in diagnostics, therapy, as well as combined therapy and diagnostic approaches by increasing the acoustic reflectivity, ultimately leading to an increase in brightness and the creation of a clearer image [247,248]. Nanosilver has been intensively used in several applications, including diagnosis and treatment of cancer and as drug carriers [249,250,251]. Nanosilver was used in combination with vanadium oxide in battery cell components to improve the battery performance in next-generation active implantable medical devices [250]. Recently, silver has been used to develop silver-based biosensors for the clinical detection of serum p53 in head and neck squamous cell carcinoma [252]. In addition, it has been explored for the location of cancer cells, and can absorb light and selectively destroy targeted cancer cells through photothermal therapy [253].

## 6. Mechanism of the Anti-Cancer Activity of AgNPs

**Figure 8:**
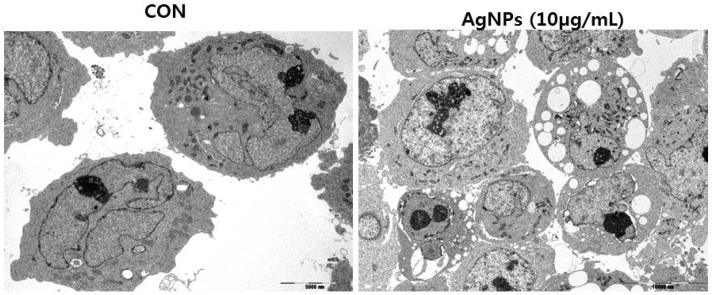
Biologically synthesized AgNPs using *Bacillus* species induce accumulation of autophagolysosomes in human ovarian cancer cells.

The next interesting aspect of silver is to find out the mechanism of AgNP-induced apoptosis in cancer cells. In this context, AshaRani et al. [254] investigated the cellular and molecular mechanisms of nanoparticle-induced effects using normal human lung cells IMR-90 and human brain cancer cells U251. They found that AgNPs were capable of adsorbing cytosolic proteins on their surface that may influence the function of intracellular factors, and that they can regulate gene expression and pro-inflammatory cytokines. Foldbjerg et al. [255] addressed the interesting aspect of cellular transcriptome analysis upon exposure of human lung epithelial cell line A549 using microarray analysis. The results from this study exhibited that AgNPs could alter the regulation of more than 1000 genes. Among several genes, metallothionein, heat shock protein, and histone families were significant [255]. Recently, autophagy-induced cell death has been another identified mechanism for the anti-cancer activity of AgNPs. Autophagy induced by nanoparticles is a critical cellular degradation process, and elevated autophagy could promote cell death [256]. Our recent findings show that AgNPs are capable of inducing autophagy through the accumulation of autophagolysosmes in human ovarian cancer cells (Figure 8). Therefore, autophagy can have a dual function; at lower levels, it can enhance the cell survival, and at elevated levels, it can cause cell death. Therefore, the use of autophagy inhibitors or autophagy protein-5 (ATG5)—small interfering RNAs (siRNA) enhanced AgNPs induced cell death in cancer cells.

The antitumor activity of AgNPs was significantly enhanced in B16 mouse melanoma cells by the inhibition of autophagy using wortmanin [256]. One of the most important mechanisms of the toxicity of AgNPs is that excessive levels of intracellular ion concentration increases the production of ROS, which is produced by cellular oxygen metabolism [15,68]. On other hand, uncontrolled ROS production can lead to serious cellular injuries [15], such as DNA damage and mitochondria-involved apoptotic cell death [1,257,258,259]. Recently, De Matteis et al. [260] proposed that endocytosed AgNPs are degraded in the lysosomes, and the release of Ag^+^ ions in the cytosol induces cell damages. Hatipoglu et al. [261] revealed that the cytotoxicity and genotoxicity of AgNPs depends on reaction times for the preparation of AgNPs. The conclusion drawn from this study suggests that AgNP seeds were the major source of toxicity. Gurunathan et al. [1] reported that cytotoxicity of AgNPs in human breast cancer cells MDA-MB-231 via the activation of p53, p-Erk1/2, and caspase-3 signaling, and the downregulation of Bcl-2. Importantly, AgNP-mediated apoptosis was a p53-dependent pathway. On the other hand, Zuberek et al. [262] demonstrated that AgNPs not only induced oxidative stress, but also indicated the influence of energy supply from glucose availability in the media. They showed evidence by growing HepG2 cells in two different media with high (25 mM) or low (5.5 mM) glucose content in the presence of 20 nm AgNPs. In this assay, they observed that AgNPs induced the dose-dependent generation of H_2_O_2_. The study suggests that lower levels of glucose are responsible for defense mechanisms. All of these studies suggest that AgNPs can induce cell death through various processes, including ROS generation, enhanced leakage of lactate dehydrogenase, upregulation of apoptosis and autophagy genes, endoplasmic reticulum stress, mitochondrial dysfunction, activation of caspases, and DNA damage.

## 7. Therapeutic Approach for Cancer Treatment Using AgNPs

The application of AgNPs in cancer is divided into diagnostic and therapeutic purposes. Several laboratories have addressed the enhancement of the therapeutic usage of AgNPs as nanocarriers for targeted delivery, chemotherapeutic agents, and as enhancers for radiation and photodynamic therapy. Here we summarized the possible therapeutic approaches for cancer using AgNPs in cancer cell lines or animal models. For instance, Lim et al. [263] synthesized plasmonic magnetic nanoparticles to enhance MRI contrast consisting of multiple components of various nanoparticles in a single platform containing silver monolayer- gold-coated magnetic nanoparticles. These coated materials showed highly efficient killing of SKBr3 cells within 3 min of near-infrared laser at the relatively low exposure of 12.7 W/cm at 808 nm. To address the efficiency of photothermal therapy, Huang et al. [264] designed an aptamer-based nanostructure which combines the high absorption efficiency of Au–Ag nanorods showing excellent hyperthermia efficiency and selectivity. The combination of AgNPs with ligands strongly influences the toxicity and cellular uptake into the cells.

Recently, photo-based nanomedicine has gained much importance for cancer treatment among several approaches [265]. Khlebtsov et al. [266] developed multifunctional NPs which significantly induced cell death in HeLa cervical cancer cells. Wang et al. [267] developed folic acid (FA)-coated AgNPs with an average size of 23 ± 2 nm showing excellent receptor-mediated cellular uptake; with this compound (FA-AgNPs), they conjugated the chemotherapeutic drug doxorubicin (DOX) by electrostatic bonding. DOX was released efficiently, and cell death was observed after 8 h. They concluded that AgNPs can be used as nanocarriers for desired drugs for cancer treatment. To increase intracellular uptake and cytotoxicity in lymphoma cells, Fang et al. [268] designed self-assembled polymer–doxorubicin conjugates such as NP-Im/DOX, NP-Ag/DOX, and NP-Dm/DOX (Nanoparticles (NP), guanidinium group (Ag), an imidazole group (Im), and a tertiary amine group (Dm), doxorubicin (Dox)) using three different cationic side chains with an average of 80 nm for the efficient delivery of nanocarrier. Locatelli et al. [269] developed a nanocarrier by using a simple method, in which lipophilic AgNPs entrapped into PEG-based polymeric nanoparticles containing chlorotoxin. The interesting aspect using this nanocarrier showed enhanced cellular uptake and cytotoxic effect.

**Figure 9:**
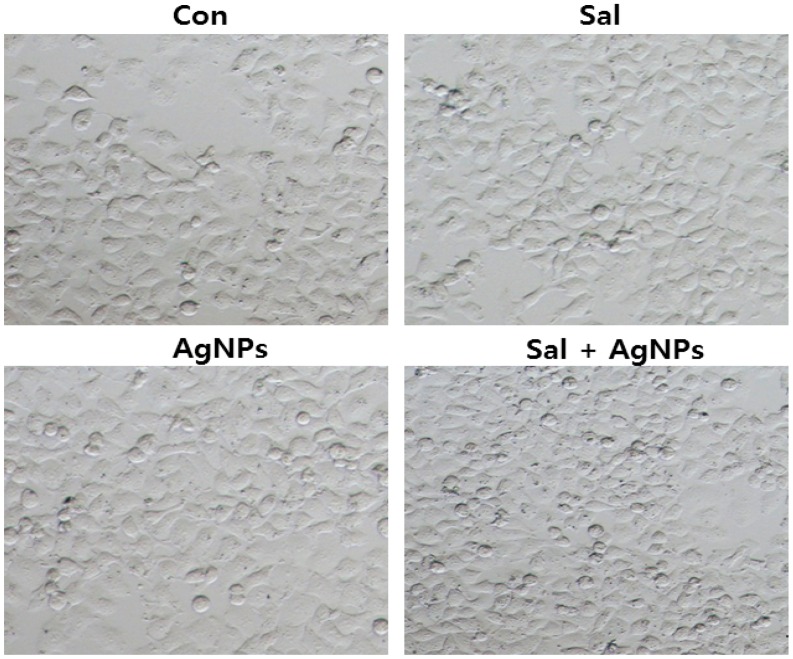
Morphological changes of the human ovarian cancer cell line A2780 after treatment with Salinimycin (Sal), AgNPs, and Sal plus AgNPs. A2780 cells were treated with Sal (3 μM), AgNPs (3μg/mL), and Sal plus AgNPs (3 μM plus 3 μg/mL) for 24 h, and the morphological changes of cells were observed under an inverted microscope (200×). The combination of Sal and AgNP induced significant morphological changes.

Recently, nanomaterials have been used for diagnosis, treatment, and prevention of cancer using photo-based therapeutic approaches [270]. The nanostructures are more capable of destroying the cancer cells than non-cancer cells at low irradiation power density [271]. In this context, Wu et al. [271] developed sensitive and specific detection aptamer-based Ag–Au shell–core nanostructure-photothermal therapy in which the nanostructures were able to target the cells with high affinity and specificity. The intra-tumoral administration of AgNPs in combination with a single dose of ionizing radiation enhanced therapeutic efficiency in C6 glioma-bearing rats [272]. Nanoparticles consisting of a silver nanoshell with a carbon core composite were significantly cytotoxic to cells in the presence of phototherapy and radiotherapy [273]. Combination of AgNPs–chitosan–para-aminothiophenol (pATP)–folic acid showed significant stability and targeted uptake in NIH:OVCAR-3 human ovarian cancer cell line. The efficient therapeutic approach was achieved by targeted cancer cell treatment with these composites [274]. Recently, Mukherjee et al. [275] used AgNPs as cancer theranostic agents; they prepared AgNPs from *Olax scandens* leaf extract and prepared AgNPs showing anticancer activity against various types of cancer cells, including A549, B16, and MCF7. Furthermore, they observed a bright red fluorescence signal from AgNPs, which can be exploited for localized drug delivery into the cancer cells. Considering the literature, AgNPs are not only used as drug delivery devices but they also serve as drugs; therefore, they are used for therapeutics [276]. AgNPs are well known antibacterial agents, and they also enhance the tumor-killing effects of anticancer drugs [276]. The combination of chemotherapeutic agents with nanoparticles is a developing effective approach for the eradication of cancer, in which they are using lower doses of drugs to reduce cytotoxic effects and increase the efficacy [277]. For example, the combination of Platinol (cisplatin) and Navelbine (vinorelbine) showed better efficiency in non-small cell lung cancer [278]. Combination of CPX-351 in a liposomal NP with cytarabine and daunorubicin showed better efficacy in the treatment of acute myeloid leukemia [279]. Similarly, the combination of salinomycin (Sal) with AgNPs derived from *Typha angustifolia* plant extracts showed a synergistic cytotoxic effect in human ovarian cancer cells (Figure 9).

**Figure 10:**
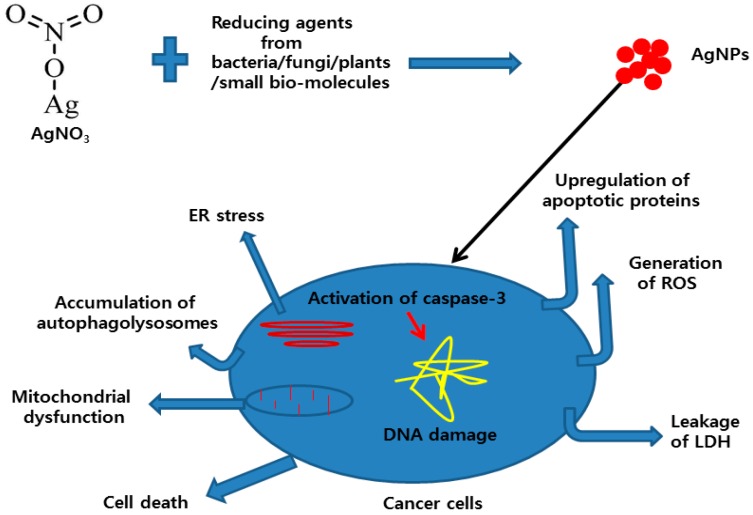
The possible mechanisms of AgNP-induced cytotoxicity in cancer cell lines. Endoplasmic reticulum stress(ER), lactate dehydrogenase (LDH), reactive oxygen species (ROS).

Altogether, published literatures suggest that the AgNPs is a suitable promising agent to inhibit the growth of cancer cells via various mechanistic approaches. The hypothetical mechanism is shown in Figure 10. 

## 8. Challenges for Cancer Therapy Using AgNPs

Nanomedicine is as one of the fast developing and promising strategies to combat cancer using metallic nanoparticles. Current treatment for cancer, such as chemo- and radiation therapy, has limitations due to unexpected drug-associated side effects, lack of specificity of low drug concentrations at the tumor target site, and the development of chemoresistance [280,281]. Nanoparticle-mediated therapy is the best, most suitable, and alternative therapeutic strategy in cancer therapy. Nanoparticles (NPs) have the ability to target through passive or active targeting of particular diseased cells or tumor tissues by the encapsulation of therapeutic agents with nanoparticles, and they have been used as drug delivery systems [282]. Although many nanoparticle-mediated strategies have been developed, heterogeneity of the tumor and its stroma is a significant challenge for nanotechnologists and clinicians to come up with specific formulations to precisely target specific cancer cells. To achieve higher specificity, reduction in toxicity, biocompatibility, safety, better efficacy, and to overcome the limitations of conventional chemotherapy, using new nanoparticles in single platform-based strategies is another challenge in cancer therapy. However, there is a need to address the challenges and limitations of using nanoparticles for cancer therapy; these include physiological barriers, limited carrying capacity, enhanced permeability and retention effect (EPR), variability of nanoparticles, and regulatory and manufacturing issues [282].

## 9. Conclusions and Future Perspectives

This review comprehensively addressed synthesis, characterization, and bio-applications of silver nanoparticles, with special emphasis on anticancer activity and its mechanisms and also therapeutic approaches for cancer using AgNPs. Recently, both academic and industrial research has explored the possibility of using AgNPs as a next-generation anticancer therapeutic agent, due to the conventional side effects of chemo- and radiation therapy. Although AgNPs play an important role in clinical research, several factors need to be considered, including the source of raw materials, the method of production, stability, bio-distribution, controlled release, accumulation, cell-specific targeting, and finally toxicological issues to human beings. The development of AgNPs as anti-angiogenic molecules is one of the most interesting approaches for cancer treatment and other angiogenesis-related diseases; it can overcome poor delivery and the problem of drug resistance. Further, it could provide a new avenue for other angiogenic diseases, such as atherosclerosis, rheumatoid arthritis, diabetic retinopathy, psoriasis, endometriosis, and adiposity.

In addition, the potential use of AgNPs for cancer diagnosis and treatment is immense; to address this issue, a variety of modalities have been developed. Although various methods are available, the synergistic effects of AgNPs and antibiotics on antibacterial agents or multiple therapeutic agents on anti-cancer activity/tumor reduction are still obscure. Therefore, more studies are required to explain the synergistic effect of the two different cytotoxic agents at a single time point. These kinds of studies could provide understanding, mechanisms, and efficiency of the synergistic effect of two different agents or multiple agents; thus, they would help to develop a novel system bearing multiple components with synergistic effects for the treatment of various types of cancer. Although AgNPs have been focused on therapeutic purposes, further research is inevitable in animal models to confirm the mechanisms and to gain a comprehensive picture of biocompatibility vs. toxicity of AgNPs. Finally, if we succeed in all these studies, it would help the researchers of the nanoscience and nanotechnology community to develop safer, biocompatible, efficient cancer or anti-angiogenic agents containing AgNPs. Eventually, to ensure the biosafety of the use of AgNPs in humans, studies dealing with biocompatibility of AgNPs and their interaction with cells and tissues are inevitable. Finally, the great concern is that the developing nanotechnology-based therapy should be better than available technologies, and it should overcome the limitations of existing treatment techniques. Finally, it has to provide a safe, reliable, and viable treatment of diseases with high accuracy in a patient-friendly manner.
